# Anti-Allergic Potential of Cinnamaldehyde via the Inhibitory Effect of Histidine Decarboxylase (HDC) Producing *Klebsiella pneumonia*

**DOI:** 10.3390/molecules25235580

**Published:** 2020-11-27

**Authors:** Lorina I. Badger-Emeka, Promise Madu Emeka, Krishnaraj Thirugnanasambantham, Hairul Islam M. Ibrahim

**Affiliations:** 1 Department of Biomedical Sciences, College of Medicine, King Faisal University, Al-Ahsa 31982, Saudi Arabia; 2 Department of Pharmaceutical Sciences, College of Clinical Pharmacy, King Faisal University, Al-Ahsa 31982, Saudi Arabia; pemeka@kfu.edu.sa; 3 Pondicherry Centre for Biological Science and Educational Trust, Kottakuppam 605104, Tamilnadu, India; researchdirector@pcbsindia.com; 4 Department of Biological Sciences, College of Science, King Faisal University, Al-Ahsa 31982, Saudi Arabia; himohamed@kfu.edu.sa

**Keywords:** allergy, histidine decarboxylase, cinnamaldehyde, mast cell, *Klebsiella pneumoniae*, cytokines

## Abstract

Allergy is an immunological disorder that develops in response to exposure to an allergen, and histamines mediate these effects via histidine decarboxylase (HDC) activity at the intracellular level. In the present study, we developed a 3D model of *Klebsiella pneumoniae* histidine decarboxylase (HDC) and analyzed the HDC inhibitory potential of cinnamaldehyde (CA) and subsequent anti-allergic potential using a bacterial and mammalian mast cell model. A computational and in vitro study using *K. pneumonia* revealed that CA binds to HDC nearby the pyridoxal-5′-phosphate (PLP) binding site and inhibited histamine synthesis in a bacterial model. Further study using a mammalian mast cell model also showed that CA decreased the levels of histamine in the stimulated RBL-2H3 cell line and attenuated the release of β-hexoseaminidase and cell degranulation. In addition, CA treatment also significantly suppressed the levels of pro-inflammatory cytokines TNF-α and IL-6 and the nitric oxide (NO) level in the stimulated mast cells. A gene expression and Western blotting study revealed that CA significantly downregulated the expressions of MAPKp38/ERK and its downstream pro-allergic mediators that are involved in the signaling pathway in mast cell cytokine synthesis. This study further confirms that CA has the potential to attenuate mast cell activation by inhibiting HDC and modifying the process of allergic disorders.

## 1. Introduction

Allergic disorders are on the rise globally, creating global health concerns [1,2]. Allergy is considered an immunological disorder which develops in response to exposure either to an allergen that could be airborne or from drugs, food, or animals. The resulting immunological disorders can subsequently present in different forms, such as asthma, urticarial, allergic rhinitis, food allergies, and anaphylaxis, which is life threatening [2].

The high prevalence of these allergic disorders cuts across the developed and underdeveloped world, with a resulting deterioration in quality of life, as it is a substantial economic burden. An estimated 25 billion dollars is reportedly used annually in the United States of America (USA) for the treatment of food allergies [3]. Generally, histamine is an amine-derived autacoid implicated in the etiology of many allergic reactions and recognized as an important mediator in several associated diseases. Its release and subsequent action are via G-protein coupled receptors (H1, H2, and H4) mediating allergy and inflammation [4]. The synthesis of this histamine is via the decarboxylation of histidine by a ubiquitous enzyme, histidine decarboxylase (HDC), stored in the basophils and mast cells in granules [5]. The degranulation of the mast cell is induced by the activation of antigen-specific IgE receptor, Fcε receptor I (FcεR1), on the surface of the mast cell [6]. The IgE-dependent degranulation of mast cells is induced by HDC, releasing numerous allergic mediators of which histamine is the main agent. Apart from being packed with granules of histamine, mast cells also contain diverse cytokines such as TNFα, IL4 CXCL-8, and IL6, which are synthesized and released when activated [7]. These released cytokines are reported to recruit other inflammatory intermediaries within the cell environment that further promote and sustain allergy and inflammation [8]. Evidence shows that mast cell activation induces the secretions of β-hexosaminidase involved in cell degranulation [9], and cytokines are reported to be part of this process as well during allergic reactions [10]. Salamon et al. [11] documented that these cytokines are implicated in the activation of phosphorylated mitogen-activated protein kinase (MAPK) pathways. The phosphorylation of the MAPK signaling cascade during IgE-stimulated antigen challenge promotes allergic reactions according to Huang et al. [12]. The components of the phosphorylated MAPK signaling pathway, MAPK p38 and extracellular-signal regulated kinase (ERK), have been reported to be involved in the pathogenesis of allergic disorders [12]. Reports, however, have shown that, when phosphorylated, they promote the release of cyclooxygenase enzyme (COX2) and nitric oxide (NO) [13]. This is because mast cell activation by the IgE-antigen complex express COX2 and stimulate the sustained production of nitric oxide (NO), hence enhancing the further release of other pro-inflammatory mediators involved in allergic disease [10]. This action further exacerbates allergic reactions. Evidence shows that the inhibition of MAPK signaling pathways will suppress the release of these allergic mediators [12]. In addition, MAPK p38 has been reported to organize the induction of HDC and consequent histamine production [14]. Evidence from the literature shows that other aforementioned released mediators of allergies also promote the induction of HDC mRNA [15].

Therefore, antigen challenge usually accompanies the induction of HDC and a consequent increase in its activity at the site of inflammation. Earlier reports [16] have demonstrated this phenomenon in HDC-deficient mice, where antigen challenge did not enhance the inflammatory activity during anaphylaxis induction. Given that multiple pathways modulate HDC expression in mast cells during allergy, the inhibition of HDC synthesis and induction could be crucial and hence represent an important treatment strategy for allergic disorders. The current drug treatment of allergic disorders acts by reducing the symptoms without modifying the disease [17]. Therefore, an agent that will modify disease initiation and progress will reduce the burden of allergy in healthcare.

It is suggested that compounds that inhibit HDC activity would be highly beneficial for treating allergy complications. Investigations [18] on screening the polyphenols in food components with HDC inhibitory potentials have suggested epicatechin gallate (ECG) and epigallocatechin gallate (EGCG) as the most potent HDC inhibitors. There have also been previous demonstrations [19] on the HDC inhibitory potential of pinocembrin as well as its anti-allergic potentials. Another plant that has been used as an anti-inflammatory is cinnamon.

The bark of cinnamon (*Cinnamomum verum*) has been used as a traditional remedy in herbal medicine for decades. It is reported to contain high amounts of cinnamaldehyde (CA) and to be a good source of proanthocyanidins, a group of plant polyphenols.

Reports have shown that cinnamon extract, besides possessing anti-inflammatory properties, exhibits antioxidant activities, as it can reduce airway inflammation and anaphylaxis in mice after antigen challenge [17]. Additionally, another report [20] has documented that CA potentially inhibits bacterial activity by interrupting membrane permeability and blocking glucose uptake, which consequently inhibits glucose utilization in bacteria. The demonstrated mechanism of action of CA in bacteria makes it a suitable candidate for reducing the bacterial generation of biogenic amines involved in allergy. Wendakoon and Sakaguchi [21] previously reported that the ethanol extract of cinnamon containing cinnamic aldehyde exhibited inhibitory activity against *Enterobacter aerogenes* produced in HDC. The biological effects of CA have been documented to significantly attenuate p38 MAPK in LPS-activated macrophages [22]. Xia et al. [23] also reported that pre-treatment with CA suppressed the p38 MAPK-JNK (Jun N-terminal kinase) pathways in IL-1β inflammation in a rat model of osteoartH.I.M.I.. The aforementioned anti-inflammatory potentials coupled with our preliminary study of the inhibition of *K. pnemoniae* HDC by CA further prompted this study. Evidence from the literature indicated that *K. pnemoniae* has a profound HDC activity and consequently an enhanced histamine production [24]. Our aim was to elucidate the anti-allergic effects of CA on stimulated basophil leukaemia RBL-2H3 cell lines and to examine the regulatory potentials of CA with respect to the synthesis of HDC, histamine, β-hexosaminidase, pro-inflammatory cytokines, and their inducers in addition to CA effects on nitric oxide (NO) activity and their expressions. An earlier report indicated that cinnamon crude extracts were found to inhibit amine formation in *Klebsiella aerogenes*, causing the arrest of amino acid decarboxylase activity [21]. In addition, this inhibited the production of biogenic amines such as histamine. In another related study, CA’s anti-inflammatory and antimicrobial properties were documented, however, specific studies on the HDC inhibitory activity of CA and its subsequent anti-allergic principle have not yet been elucidated.

In this study, we evaluate cinnamaldehyde (CA), a major component of cinnamon essential oil, as a potential anti-allergic agent and utilize computational and in vitro approaches to validate the histidine decarboxylase inhibitory activity and subsequent anti-allergic effects of cinnamaldehyde.

## 2. Results

### 2.1. Structural and Docking Study of the HDC Protein

**Figure 1:**
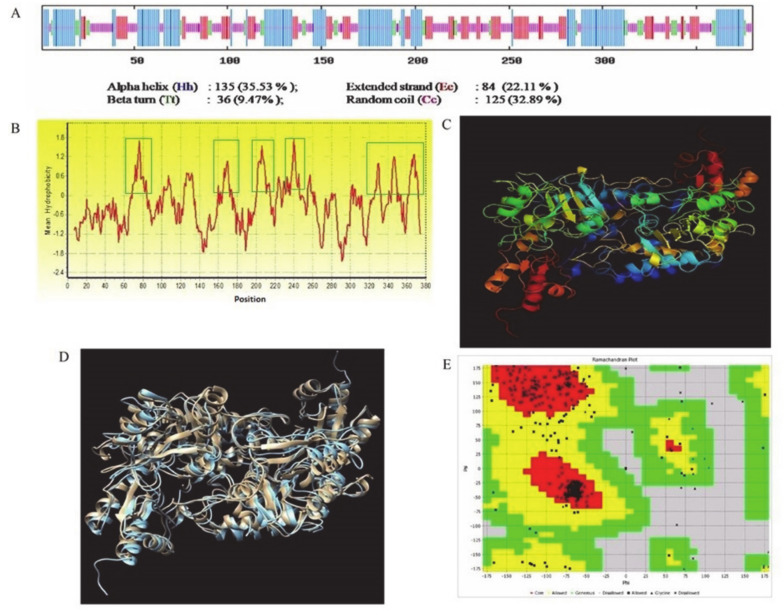
Represents the results of the predicted structural analysis of *K. pneumoniae* histidine decarboxylase protein. (**A**) Secondary structure conformation parameters of *K. pneumoniae* histidine decarboxylase based on self-optimized prediction method with alignment (SOPMA) analysis. Helices, extended strand/sheets, beta turns, and random coils are indicated with the blue colored longest lines, the red colored second longest lines, the green colored second shortest lines, and the pink colored shortest vertical lines, respectively. Most of the secondary structure of *K. pneumoniae* histidine decarboxylase is composed of alpha helix, which is followed by random coil, extended stand, and beta-turn, respectively. (**B**) Hydropathy plot of *K. pneumoniae* histidine decarboxylase shows that it is hydrophobic in nature. Green line box depicts the presence of more than 7 non-polar amino acid residues such as Glycine, Alanine, Proline, Valine, Leucine, Isoleucine, Methionine, Tryptophan, and Phenylalanine in structural topology, which increases the stability of the protein and prevents hydrolysis. More positive values indicate more hydrophobic amino acids located in that region of histidine decarboxylase (HDC) protein. (**C**) Homology-modeled three-dimensional structure of *K. pneumoniae* histidine decarboxylase, using *Methanocaldococcus jannaschii* L-tyrosine decarboxylase (pbd id 3f9tA) as the template structure. (**D**) Superimposition of the predicted structure of *K. pneumoniae* histidine decarboxylase with the template *M. jannaschii* L-tyrosine decarboxylase (pbd id 3f9tA). Superimposition of modeled histidine decarboxylase of *K. pneumoniae* to the template *M. jannaschii* L-tyrosine decarboxylase structure revealed the perfect homology among the structures. Pale orange color represents the 3D structure of the template protein, while the blue color represents the 3D structure of *K. pneumoniae* HDC superimposed on the template structure. (**E**) Ramachandran plot of the predicted *K. pneumoniae* histidine decarboxylase 3D model. Presence of the majority of amino acids in the allowed region (yellow) of the Ramachandran plot revealed the quality of the modelled 3D structure of *K. pneumoniae* histidine decarboxylase.

The microbiological model of the predicted structure of *K. pneumoniae* HDC was docked against CA in a preliminary study. The primary, secondary, and 3D structure properties; amino acid composition; Ramachandran plot analysis; and G-factor parameters are presented in Figure 1a–e and Tables S1–S4 (Supplementary Data). Secondary structural analysis using a self-optimized prediction method with alignment (SOPMA) revealed that the majority of the protein sequence is involved in an alpha helix followed by a random coil, extended stand, and beta turn, respectively (Figure 1a). The averages hydrophobicity scores were plotted on a graph with the Y-axis representing hydrophobicity scores, while the X-axis shows the position of amino acids with peaks. The amino acids shown on the surface and interior regions of the protein structure are identified as peaks above and below the midline, respectively. The hydrophobicity plot for HDC shows that hydrophilic amino acids are presumably buried in the interior of the protein, with the hydrophobic amino acids assembled on the surface of the protein. Thus, the hydrophobicity plot revealed that the HDC surface is hydrophobic in nature. Therefore, the hydrophobicity plot of the amino acid sequence shows the protein to be hydrophobic in nature (Figure 1b). The perfect imposition of the modelled 3D structure of the *K. pneumoniae* HDC (Figure 1D) with the template structure (Figure 1c) and the presence of a majority of amino acids in the favored region of the Ramachandran plot (Figure 1e) revealed the quality of the modelled structure.

**Figure 2:**
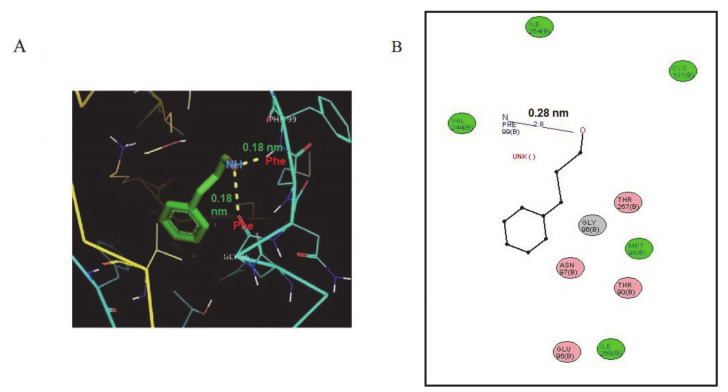
Results of the docking analysis of *K. pneumoniae* HDC protein with cinnamaldehyde (CA). (**A**) Residues of *K. pneumoniae* histidine decarboxylase docking showing amino acid interactions with CA. (**B**) In silico analysis of ligand binding with the protein structure of HDC as well as amino acid interactions at the atomic level of hydrogen with residues and with the measurement of distance between interacting molecules, as shown in (**A**) and (**B**).

A docking analysis of the predicted HDC 3D structure with CA revealed an obvious interaction with a binding energy of −5.14 kcal/mol (Table S2), indicating an interaction with the two active residues of Phe 99 (1.8 Å-[0.18 nm]) and Gly 96 (2.6 Å-[0.26 nm]). (Figure 2a, Table S4). The potential interaction between CA and HDC formed two hydrogen bonds. In Figure S1a, the docking of the product (histamine) with HDC revealed a three-hydrogen-bond interaction, and amino acids THR 94 with bond length of 1.7 Å (0.17 nm) and ASN 97 with a bond length of 2.1 Å (0.21 nm) are involved (Figure 2b). The docking of the substrate (histidine) with HDC revealed a two-hydrogen-bond interaction, and amino acids THR 94 (2.1 Å (0.21 nm)) and SER 122 (2.7 Å (0.27 nm)) are involved (Figure S1b, Table S4). The docking of pyridoxal-5′-phosphate (PLP) revealed a five-hydrogen bonding with THR`94 (2.0 Å (0.20 nm)) and GLU`95 (2.8 Å (0.28 nm)) in the A chain, while B chains have a 3.2 Å (0.32 nm) bond length for only GLU’95 (Figure S1c, Table S4).

### 2.2. Inhibition of Bacterial HDC Activity by CA

**Figure 3:**
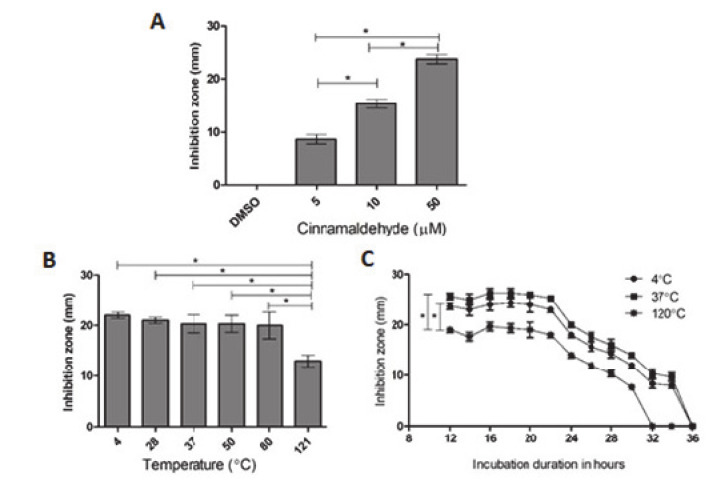
(**A**–**C**) Representative *Klebsiella pneumoniae* HDC inhibitory activity by cinnamaldehyde (CA). (**A**) Zones of inhibition, as measured on plates in millimeters (mm) after 12 h. (**B**) Effect of temperature on the CA inhibitory effects from (4–121 °C) of zones of inhibition (mm), as determined by the plate inhibitory method. (**C**) CA inhibition measured from 0 to 36 h at a concentration of 50 µΜ. Inhibitory zone was measured using a regression plot. Data are presented as the mean ± S.D. of three independent replicate experiments. Significance were expressed as * *p* < 0.05.

The potential anti-allergic activity of CA by direct interaction with the HDC downstream enzyme was tested with a microbiological model of *K. pneumoniae* as aforementioned. The results obtained using a differential plating method are presented in Figure 3a–c and Figure S2. The preliminary results showed that CA inhibited the activity of bacterial HDC after 12 h of incubation in a concentration-dependent manner (5, 10, and 50 μmole/L) compared to the DMSO-treated control (Figure 3a). Furthermore, the temperature stability study revealed that the CA inhibitory activity of HDC was effective up to 80 °C and declined at 121 °C (Figure 3c). The effect of the time factor on the inhibitory activity of thermally treated CA was examined as well. At 4 h intervals for 36 h, the inhibitory effects of CA on bacterial HDC peaked between 16 and 22 h and gradually diminished until 36 h. The results of this time course of thermally treated CA exposed for a period of 36 h using a 50 μM concentration further confirm the CA inhibitory potentials against HDC activity.

### 2.3. CA Cytoprotective Role against IgE-Mediated Response and HDC Activity

**Figure 4:**
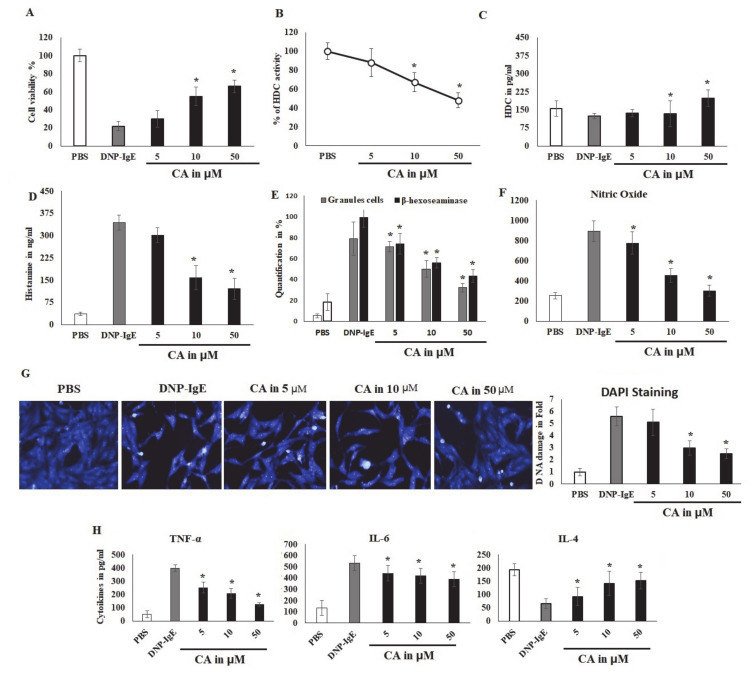
Effects of cinnamaldehyde (CA) on the cell viability and HDC activity. (**A**) Viability of untreated RBL-2H3 cells incubated with CA for 24 h quantified by -(4,5-dDimethylthiazol-2-yl)-2,5-diphenyltetrazolium bromide (MTT) assay compared with IgE sensitization controls. (**B**,**C**) An analysis of the inhibition of HDC activity by CA treatment and the quantification of HDC using RBL-2H3 cells in in vitro IgE-mediated allergic reactions with various concentrations of CA. (**D**) The effect of CA on the percentage of histamine quantification in RBL-2H3 cells values, expressed as ng/mL of histamine in the medium. (**E**) Analysis of the CA effect on the percentage of cellular granules and degranulation markers (Hexoseaminidase) in stimulated RBL-2H3 cells examined and counted under a high-power phase contrast microscope. (**F**) Nitric oxide quantification using the reduction of greiss reagents and values are observed at 402 nm. (**G**) Anti-apoptotic results on the effects of CA on DNA integrity and strand degradation imaging using florescence 4',6-Diamidino-2-Phenylindole (DAPI) staining dye. DNA damage was quantified with arbitrary units of silver blue color and recorded as arbitrary units. (**H**) The effect of CA on the cytokine expression in DNP-IgE stimulated RBL-2H3 cell lines. Cytokines such as TNF-α, IL-6, and IL-4 were quantified and expressed as pg/mL. Data are shown as the mean ± S.D. of representative experiments out of three independent experiments performed in triplicate and yielding similar results. (* *p* < 0.05).

The results of the effects of CA on the cell viability of untreated basophilic leukemia RBL-2H3 cells and DNP-BSA sensitized RBL-2H3 cells were studied as presented in Figure 4a–e. There was a significant decrease (*p* < 0.05) in the cell viability by 50% at a concentration of 100 μM, indicating that CA had the capacity to suppress the viability and proliferation of basophilic leukemia RBL-2H3 cells. However, lower concentrations only produced 75% cell viability reductions (Figure 4a). The effect of a narrow range of concentrations (5–50 μM) of CA was examined on DNP-BSA-sensitized IgE-challenged RBL-2H3 cells. Cells challenged with DNP-IgE produced profound cell death, whereas exposure to various concentrations of CA ameliorated these effects and subsequently enhanced the cells’ viability and survival (Figure S3). The exposure of CA for 10 min prior to sensitizing RBL-2H3 cells with IgE revealed a reversal of DNP-BSA-stimulated damage to the integrity and adhesion of the cells (Figure S3). The results of the prediction of the inhibitory potential of CA on HDC activity on sensitized RBL-2H3 in the presence of histidine showed a 40% inhibition in a concentration-dependent manner, which was significant (*p* < 0.05) (Figure 4b). In the same fashion, the effect of CA on the histamine levels was also explored, due to the fact that histamine is synthesized by the enzymatic action of HDC. Although the HDC level was not altered even by lower concentrations of CA treatment, it was, however, significantly (*p* < 0.05) elevated in DNP-BSA stimulation mast cells at a 50 µM concentration (Figure 4c). This result confirms the view of the inhibitory role of CA on HDC without affecting its protein level.

### 2.4. CA Attenuates Antigen Induced Mast Cell Response

The effect of CA was also examined on other pro-allergic reactions in IgE-antigen-sensitized RBL-2H3 cells by studying the cytoplasmic granules. The granulation leads to the release of stored histamine; hence, the histamine level in ng/mL was quantified. The results showed that CA significantly (*p* < 0.05) reduced the release of histamine by controlling its granulation (Figure 4d). It was also observed that stimulating the cells with DNP-BSA significantly increased the percentage of granule content in the cells. However, when the cells where pre-incubated with CA at concentrations of 5, 10, and 50 μM, the granulation levels were reduced markedly (*p* < 0.05) (Figure 4e), since cells are typically primed for degranulation as cytoplasmic granulation increases. In this regard, we quantified the effect of CA on known markers of mast cells and basophil degranulation, β-hexoseaminidase. The results showed that CA also significantly (*p* < 0.05) suppressed the synthesis of β-hexoseaminidase. On a parallel note, cells stimulated with DNP-BSA were observed to augment the production of β-hexoseaminidase and consequently increased its synthesis, which CA attenuated in concentrations of 5, 10, and 50 μM (Figure 4e). The predominant anti-inflammatory marker nitric oxide was negatively regulated by CA in IgE-sensitized mast cells (Figure 4f). This is because allergic reactions are mediated by nuclear alteration and degradation as part of the apoptotic process of inflammation. The nuclear modification was analyzed by DAPI nuclear staining, and it was found that CA controlled the nuclear damage and reversed the DNP-IgE-mediated DNA alterations (Figure 4g).

### 2.5. Modulatory Effects of Pro-Inflammatory Mediators

The effects of CA on cytokine-mediated allergic reactions in mast cells are shown in Figure 4h. Antigen-mediated stimulation activates allergic responsive cells, causing them to infiltrate and initiate the process of inflammation. In this study, CA reduced the secretions and release of these extracellular inflammatory cytokines, including TNF-α and IL-6. Thus, CA reversed these pro-inflammation cytokines and increased IL-4, an anti-inflammatory marker in a concentration-dependent manner.

### 2.6. Effects of CA on In Vitro Allergic Markers

**Figure 5:**
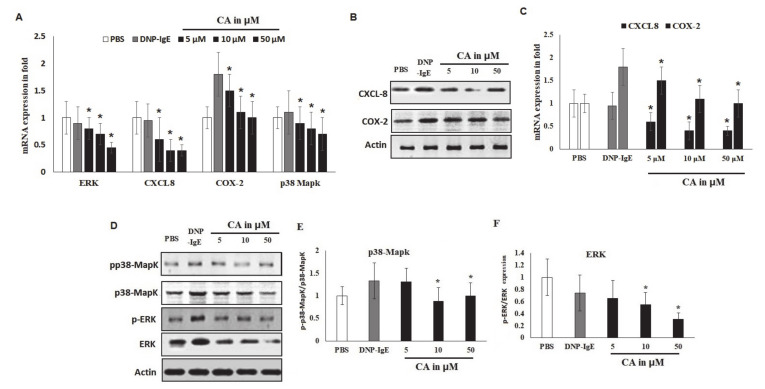
Cinnamaldehyde (CA) effect on allergic markers using in vitro models. (**A**) PCR mRNA quantification of ERK, CXCL-8, COX-2, and p38-MAPK markers in DNP-IgE-sensitized mast cell lines with β-Actin used as an internal control. (**B**,**C**) Western blot analysis by an immunoblot of CXCL-8 and COX-2 recorded with a LICOR signal recorder. The blot was segregated with ERK and p38 MAPK with and without the phosphorylated form. (**D**–**F**) p-p38 MAPK and p-ERK protein estimation was noted in Dinitrophenyl (DNP)-IgE-sensitized mast cell lines, with data presented as the results of three independent replicate experiments, mean ± S.D. Significant difference is represented as * *p* < 0.05.

The results showed that CA selectively suppressed the mRNA levels of ERK, CXCL-8, and COX-2 at 50 µΜ (Figure 5a–d). However, although CA decreased the mRNA levels of p38 MAPK, the difference was not significant. Importantly, the phosphorylation of p38 was not marked compared to that of ERK. The protein levels of these markers were significantly (*p* < 0.05) suppressed by the CA treatment. It was observed that this effect was more potent on ERK and CXCL-8 markers than other tested markers. Therefore, COX-2 and p38-MAPK reductions reveal the reversal of the activation of inflammatory markers at 10 and 50 μM (Figure 5e,i).

Cinnamaldehyde (CA) effect on allergic markers using in vitro models (A–D) PCR mRNA quantification of ERK, CXCL-8, COX-2, and p38-MAPK markers in DNP-IgE-sensitized mast cell lines with β-Actin used as an internal control. (E) Western blot analysis by an immunoblot of ERK, CXCL-8, COX-2, and p38-MAPK recorded with a LICOR signal recorder. The blot was segregated with ERK and p38 MAPK with and without phosphorylated form. (F–I) ERK, CXCL-8, COX-2, and p38-MAPK protein estimation was noted in DNP-IgE-sensitized mast cell lines, with data presented as the results of three independent replicate experiments. Significant difference is represented as * *p* < 0.05, and data are presented as the mean ± S.D. of the three independent replicate experiments. 

## 3. Discussion

Cinnamaldehyde is a bioactive phenylpropanoid compound and a major component of cinnamon oil isolated from the bark of cinnamon tree. Several studies have confirmed that CA has antimicrobial activity against bacteria and fungi [25], especially against *K. Pneumoniae* [26]. Crude essential oils containing components of cinnamaldehyde have been used to treat diseases associated with inflammation as traditional herbal medicine [27]. Nasal spray prepared from cinnamon tree bark has been shown to ameliorate allergic rhinitis in patients and are reported to be non-toxic [28]. Relief of allergic rhinitis by extracts of cinnamon tree bark was seen in the attenuation of histamine release and the decrease in lipid mediators that induce and exacerbate allergic reactions [28]. Documented evidence shows that mast cell activation mediates both the early-phase and late-phase hypersensitivity reactions. Both phases are predicated in the activities of HDC in the release of histamine from the mast cells along with other inflammatory mediators. Therefore, the control of HDC activities in the regulation of mediators will help to modify the allergic inflammatory disease process. Compounds that inhibit HDC activities will represent a new target for the treatment of allergic diseases. A wide range of laboratory models have been adopted to study the anti-allergic potential of biomolecules. *Klebsiella pneumoniae* has previously been utilized as a preliminary microbiological model to study the HDC inhibitory potential and anti-allergic properties [19]. In addition, CA has been reported to inhibit completely *K. pneumoniae* activity in vitro, and it could potentially reflect on its ability to synthesize amino acids [26]. Furthermore, experimental evidence has shown that crude extract of cinnamon containing cinnamic aldehyde was very effective in diminishing 90% of HDC activity isolated from *K. aerogenes* [21]. This evidence is linked to other studies that report that *K. pneumoniae* has a strong histidine decarboxylase activity and consequently an enhanced histamine production capacity [28]. The observations showed that *K. pneumoniae* could be utilized as a potent microbiological model to screen HDC inhibitory of other natural molecules. Due to the non-availability of structural details of *Klebsiella pneumoniae* HDC enzymes, its characterization was undertaken. Hence, in the present study, we adopted a computational approach for the prediction and validation of *K. pneumoniae* HDC using *Methanocaldococcus jannaschii* L-tyrosine decarboxylase (pbd id 3f9t) as a template structure. Hence, this investigation adopted PS2, an automatic homology model, to develop 3D structures of plant and animal proteins, as has been reported previously [29,30]. Similar to our study, Baker [31] reported a 3D structure of pig dopa decarboxylase (DDC) using human HDC as a template by adopting comparative modelling techniques. In another research [32], *Klebsiella planticola* HDC was built based on homology modelling with human PLP-dependent glutamate decarboxylase that shared just 24% protein sequence identity. Similarly, the *K. pneumoniae* HDC modelled in the present study revealed 25% protein sequence identity with the *Methanocaldococcus jannaschii* L-tyrosine decarboxylase (pbd id 3f9t) template structure. According to Sandmeier et al. [33], bacterial HDC enzymes are homologous to both mammalian aromatic L-amino acid (and Dopa) decarboxylase (DDC) and HDC. We then utilized *K. pneumoniae* HDC as the primary tool to analyse the anti-allergic potential of CA. The results from our docking investigation showed that CA could bind to amino acids (PHE`99 and GLY`96) closer to the Pyridoxal-5′-phosphate (PLP) binding site (GLU`95) in HDC.

This could lead to conformational change in HDC’s PLP binding site and the subsequent inhibition of enzymatic activity. Epigallocatechin gallate (EGCG), a natural compound with promising anti-inflammatory activity, has been reported to bind mammalian HDC, changing the PLP conformation inside its catalytic site, thus inhibiting enzymatic activity by blocking its reaction with histidine [34,35]. Other researchers [36] have applied quantum mechanics (QM) and molecular mechanics (MM) simulations and found that the PLP-histidine complex was located in the HDC catalytic site. In the presence of natural substrates or synthetic analogues, local changes in the active site of HDC have been reported to affect its confirmation and enzymatic stability [37]. Although histidine and histamine do not affect HDC activity, PLP was reported to increase the specific enzymatic activity of bacterial HDC without affecting its gene expression [38]. In our preliminary study on the effect of CA on HDC isolated from the Gram-negative bacteria *Klebsiella pneumoniae*, we observed a dose-dependent inhibitory effect. Positive results from the computational study gave us the impetus to further confirm the HDC inhibitory potential of CA using an in vitro approach. The in vitro toxicity study using RBL-2H3 cells revealed that CA, at a concentration of >100 μM, was non-toxic, which is consistent with other reported in vitro studies using mucosal-type bone-marrow derived mast cells [39]. It was also found that CA inhibited HDC activity in a dose-dependent manner without affecting the level of HDC enzymes. Our data also indicated that exposure to CA suppressed β-hexosaminidase release, histamine synthesis, and associated cellular degranulation. Although it had been reported [40] that CA decreased the release β-hexosaminidase from the mast cells, this is the first report to reveal the HDC inhibitory potential of CA in histamine-producing cell lines. Another research [41] reported that histamine could be responsible for phospholipase C activation and the subsequent release of intracellular Ca^2+^. Decreases in the intracellular calcium levels were reported to suppress the degranulation of mast cells, and its regulation was suggested to be an effective target in allergic inflammation [42].

The use of cinnamon bark extract as an intranasal spray in seasonal allergic rhinitis patients has been reported to be safe and without any toxic effects by Walanj et al. [28]. In our current study, different concentrations of CA showed less toxicity to RBL-2H3 cells. The exposure of these cells to CA displayed the inhibition of cell viability, proliferation, and induced apoptosis in a dose-dependent manner in non-sensitized and sensitized RBL-2H3 cells with DNP-IgE. Such proliferation and migration are common features of the allergic inflammatory process, which involves mediator releases [22]. Similar results have also been found using human aortic smooth muscle cells by Jin and Kim [43].

Generally, cytokines play a central role in the pathogenesis of allergic inflammation [44], with related studies documenting that both pro-inflammatory cytokines and chemokines, including TNF-α, IL-6, NO, and CXCL8, contribute to the pathogenesis and exacerbation of allergic asthma [45]. The present study hereby reports that the TNF-α and IL-6 activity was significantly suppressed in antigen-challenged DNP-IgE RBL-2H3 cells in a concentration-dependent manner. Our experimental finding is in agreement with the work of Roth-Walter et al. [46], who observed a reduction in TNF-α in human peripheral blood mononuclear cells (PBMC) after incubation with different concentrations of CA. The level of IL-6 has been suggested to correlate with the degree of allergic inflammation through IgE production [47]. Previous studies have postulated that targeting and suppressing these cytokines could provide precision treatment in patients with allergic asthma [45], because evidence shows that allergic inflammatory cytokines induce the expression of HDC, even in non-mast cells [4]. The effects of NO on mast cell functions, such as activation and mediator release, has been well documented [48]. According to Forsythe et al. [48] evidence show that mast cells express nitric oxide synthase (NOS) and therefore a source of endogenous NO Therefore, mast cell activation causes an additional release of NO, which is synthesized spontaneously by the control of inducible nitric oxide synthase (iNOS) [9]. According to a recent study [49], NO elaboration is associated with the pathogenesis and progression of asthma. In the present investigation, CA was found to decrease the release of NO in antigen-challenged RBL-2H3 cells. Given that NO is implicated in this role, a reduction in its synthesis may in addition represent a treatment strategy in the control of allergic asthma. In addition, the anti-inflammatory effects of IL-4 have been reported and demonstrated in a rat artH.I.M.I. model, showing a reduction in the artH.I.M.I. index [50]. It also showed reduced cartilage destruction in a rat instability-induced experimental osteoartH.I.M.I. model. [51]. Our study revealed a dose-dependent increase in the activity of IL-4, confirming an earlier observation reported by Ulker et al. [52]. Kedong et al. [53] reported that the effectiveness of IL-4 in inhibiting inflammatory pathways in chondrocytes by decreasing the release of IL-6 and IL-8 was reduced significantly. Mast cell activation also results in the stimulation of MAPK pathways, leading to the phosphorylation of p38/ERK, cytokine release, and degranulation [54]. The activation of the p38 MAPK pathway contributes to inflammation, apoptosis and cell differentiation. Phosphorylation of MAPKs causes the inflammatory mediators’ production and promotes an allergic inflammatory response. MAPK pathway activation promotes a number of inflammatory mediators, including COX-2 and iNOS [12]. This observation shows that MAPK signaling is important in the mediation of allergic inflammation. Therefore, the inactivation of MAPKs subsequently decreases the allergic inflammatory response [12]. The inhibition of phosphorylation and the activation p38/ERK has been shown to reduce the transcription and expression cytokines [55].

We therefore examined the activities and expressions of both the phosphorylated and protein forms of MAPK p38/ERK, including CXCL-8 and COX 2. The present study also shows significant downregulated p38 and ERK, including CXCL-8 and COX 2. The present study also shows significant downregulated expressions of chemokines and pro-inflammatory markers. Both the mRNA and protein levels of p38, ERK, CXCL-8, and COX 2 were downregulated. Similar findings were documented [56] using cinnamon extracts and their derivatives. Suppressing the p38 and ERK pathways suggests that their regulatory roles in allergic inflammation could be modified by CA. ERK is involved in the regulation of CXCL8 expression; the reduced expression of ERK can explain the influence of CA on the expression of other pro-inflammatory cytokines [40]. Documented evidence shows that ERK expression is significantly increased in the airway tissue in asthmatic patients [57]. A previous study demonstrated a significant increased activity of ERK in the lungs of asthmatic mice compared to normal mice used as control [58]. Our study hereby confirms the evidence for the potential of CA in the treatment of various allergy-associated disorders, as has also been documented [27,59].

## 4. Materials and Methods

### 4.1. Computation of Primary and Secondary Parameters of HDC Protein

The NCBI Acc No. WP_016529303 protein sequence encoding the full length for *Klebsiella pneumoniae* histidine decarboxylase (HDC) gene was used for the primary bioinformatics analysis. Moreover, using ProtParam (http://www.Expasy.ch/tools/protparam.html), the following primary parameters of *K. pneumoniae* HDC, molecular weight, theoretical isoelectric point (pI), amino acid composition, extinct coefficient, estimated half-life, instability index, aliphatic index, and grand average of hydropathicity were determined computationally. Using the self-optimized prediction method with alignment (SOPMA) (http://npsa-pbil.ibcp.fr/cgibin/npsa_automat.pl?page=/NPSA/npsa_sopma.html), the secondary structural conformational parameters were also computed.

### 4.2. The Prediction and Evaluation of the Three-Dimensional Model

The online protein prediction tool, PS2, on a protein prediction server (http://www.ps2.life.nctu.edu.tw/) was used to predict the 3D structure of *K. pneumoniae* HDC as previously described [29]. The analysis of the predicted models was based on the DOPE score, while the quality of the sterochemical model was analyzed with PROCHECK using a Ramachandran plot on the SAVES server, as has been previously performed [60]. The predicted *K. pneumoniae* HDC model superimposition with the *Methanocaldococcus jannaschii* L-tyrosine decarboxylase (pbd id 3f9tA) template was carried out with SALIGN (http://salilab.org/salign) and visualized using the UCSF Chimera package [61]. The predicted protein structure activity sites were searched with Q-site Finder, where a putative ligand could bind and optimize its van der Waals interaction energy, as previously described [62].

### 4.3. Molecular Docking of Cinnamaldehyde

The chemical structure of the cinnamaldehyde ligand was retrieved from the PubChem compound database (http://www.ncbi.nlm.nih.gov/search), and the retrieved chemical structure was converted from sdf to pdb format for analysis using the PyMOL software. The docking studies were carried out using the AutoDock tools (ADT) v1.5.4 and AutoDock v4.2 programs.

### 4.4. An Assay of HDC Activity

*Klebsiella pneumoniae* was used as the source of HDC for preliminary investigations to examine the inhibitory effects of CA. Previously described methods [19,63] were used for the quantification of the activity of HDC and were carried out by differential plating methods. An overnight cultured growth of *Klebsiella pneumoniae* was inoculated and spread on selective media containing tryptone, yeast extract, L-histidine·2HCl, NaCl, CaCO_3_, and bromocresol purple at pH 5.3 (Himedia, Mumbai, India).

Following the aforementioned procedure, dimethyl sulfoxide (DMSO) was diluted by a sterile differential bacterial broth method, yielding a final concentration of 0.1%. This was then used for the dilution of CA (Sigma Aldrich, St. Louis, MO, USA) to obtain final concentrations of 5, 10, and 50 µmole/L. Twenty-five microliters of dissolved CA were inoculated into agar wells and incubated at a temperature of 37 °C for 12 h (hours). The zones of inhibition measured in millimeters (mm) were used in quantifying the activity of HDC. Furthermore, aliquots of CA (50 µmole/L) were used for thermal stability investigations. These were incubated at temperatures of 4, 28, 37, 50, and 80 °C for 24 h, after which the resultant treated CA (50 µmole/L) on HDC inhibitory effects was measured and compared with untreated CA. The activity of HDC was measured at time intervals of 2 h, from 12 to 36 h, for the evaluation of the time course by introducing thermally threated CA (50 µmole/L) into agar wells.

### 4.5. Cell Viability and Cytotoxic Study Using Stimulated RBL-2H3 Cells

The basophilic leukemia RBL-2H3 cell line obtained from American Type Culture Collection, ATCC, VA, USA, was used for the investigation. Cells were incubated anaerobically (5% CO_2_) at a temperature of 37 °C on Dulbecco’s modified Eagle’s medium (DMEM) which had been supplemented with fetal bovine serum (10%), glutamine (0.0%), and 1% penicillin/streptomycin (Gibco, Rockville, MD, USA). Cytotoxicity was ascertained by seeding RBL-2H3 cells at 1 × 10^4^ cells in a 96-well plate and incubating them with CA at 1, 5, 10, or 50 µmole/L for 10 min. The method used for the sensitization of cells and the determination of the viability of cells by a-(4,5-dDimethylthiazol-2-yl)-2,5-diphenyltetrazolium bromide (MTT) assay is as previously described [19,64], with the cell viability measured at 540 nm calorimetrically over the last 4 h.

### 4.6. An Assay of Inhibitory Effects of HDC

A mammalian HDC enzyme source was prepared as previously described by suspending DNP-BSA-stimulated RBL-2H3 cells (1 × 10^8^) in 4 mL of non-denaturing lysis buffer while incubating on ice, and then lysed by an ultrasonic disruptor [19]. For enzyme kinetic analysis, the methods of Kanki et al. [65] was used, where HDC containing lysate with and without CA (50 µmole/L) was incubated at 37 °C for 2 h. The degree of HDC activity was measured at 405 nm. The reaction initiation and completion accompanying inhibition were quantified spectrophotometrically at 405 nm. The percentage inhibition was calculated as follows: inhibition % = (1 − [A − B]/C) × 100, where A = inhibition plus enzyme absorbance; B = inhibitor and without enzyme; C = enzyme and without inhibitor.

### 4.7. Determination of Nuclear Integrity, Cell Granulation and Apoptosis

Using 5, 10, or 50 µL of either DMSO or CA, RBL-2H3 cells (2 × 10^4^) were pre-treated for 10 min. This was followed by 24 h of sensitization with anti-DNP-IgE and by DNP-BSA for another 24 h [19]. Granules containing cells were counted in four microscopic fields when examined using a high-power phase microscope. The process of the quantification of β-hexoseaminidase is as previously described [66], and the density of the obtained color was measured at 405 nm calorimetrically. The DAPI staining level using a fluorescent microscope was used to ascertain the nuclear integrity and damage of the sensitized and stimulated RBL-2H3 cells.

A cell death detection kit from Roche Diagnostics and DAPI staining were used for quantifying apoptotic cells in situ using CA incubated for 18 h according to the manufacturer’s guidelines and analyzed using fluorescence microscopy.

### 4.8. Quantification of Nitric Oxide (NO) and Immunoblot

The quantification of extracellular NO was carried out spectrophotometrically at 540 nm with a Griess reagent, as described previously [67]. For this, RBL-2H3 cells (2 × 10^4^) were pre-incubated with either DMSO or CA at 5, 10, or 50 µmole/L for 10 min, and were sensitized with anti-DNP-IgE followed by DNP-BSA over a 24 h period.

An ELISA (Enzo Life science Inc, Lausen, Switzerland) was then used to quantify the supernatant concentrations of histamine, TNF-α, IL-4, and IL-6 (Genway, San Diego, CA, USA). Using Western blot, proteins of ERK, CXCL-8, COX-2, and p38Mapk were quantified, while targeted proteins were detected using a specific RIPA lysis buffer and fractionated using SDS-page. Specific rabbit polyclonal antibodies were used to detect these targeted proteins, with the β-actin quantified for reference (Tocris Bioscience, Bristol, UK). Using Image LiCor (Licor, Lincoln, NE, USA), the intensities of the bands were quantified.

### 4.9. Analysis by Real-Time PCR

Real-time PCR analysis was carried out with the Stratagene Agilent Real-time PCR system (Agilent Technologies, CA, USA) for total RNA extraction according to the manufacturer’s guidelines at cycling conditions at 95 °C for 5 min, 40 cycles at 95 °C for 15 s, and 60 °C for 1 min. The primers shown in Table 1 and SYBR Green (GeNetbio) were used for the quantification of the mRNA of iNOS and COX-2 and the endogenous control β-actin with the fold change in target mRNAs calculated using the comparative ΔΔCt method [68].

### 4.10. Statistical Analysis

Data were analyzed with SPSS statistical software version 23, and the values were expressed as mean ± SD determined from experimental triplicates. Significance differences between the treatment groups were statistically determined by a one-way analysis of variance (ANOVA), followed by Tukey’s post hoc test. Statistically significant levels were considered at *p* < 0.05.

## 5. Conclusions

We have demonstrated that CA potentially inhibits both bacterial and RBL-2H3 cells’ HDC activities. The study also revealed that CA attenuates the degranulation of mast cells, thereby decreasing β-hexosaminidase release and attenuating histamine synthesis in IgE-antigen-stimulated RBL-2H3 cells. The exposure of these cells to CA also downregulates the expressions of p38, ERK, CXCL-8, and COX-2, the downstream targets involved in the signaling pathways in mast cell cytokine activation. Therefore, since CA modifies the activity of mast cells by suppressing the release of mediators associated with allergic inflammation via HDC inhibition, our results suggest that it could be a potent nutraceutical that can be utilized as a safe anti-allergic agent.

## Figures and Tables

**Table 1:** Details of the sequences of real-time PCR primers.

Primer Name	Forward	Reverse	Product Size
MAPK-6	CCATCTCAAGCCACCCTTTCCA	CTCCGAGAACTGACAATCGTGG	135
ERK-1	TACAAGCTTAGCTCGGCCTAT GACCACGTG	TACGAATTCGGCTTTAGATCT CGGTGGAGC	264
CXCL-8	ATGACTTCCAAGCTGGCCGTGGCT	TCTCAGCCCTCTTCAAAAACTTCTC	141
COX-2	GAATCATTCACCAGGCAAATTG	TCTGTACTGCGGGTGGAACA	149
β-ACTIN	GATGGCCACGGCTGCTTC	TGCCTCAGGGCAGCGGAA	165

## Supplementary Materials

The following are available online. Figure S1: In silico docking of Histamine, Histidine, and PLP for proof of evidence on the binding site of amino acid residues. Figure S2: Differential screening medium for histamine production inoculated with *Klebsiella pneumoniae*. Figure S3: Microscopic examination of cell viability and survivability effect of CA on RBL-2H3 cells. Table S1: *Klebsiella pneumoniae* histidine decarboxylase primary structural properties. Table S2: *Klebsiella pneumoniae* histidine decarboxylase Amino acid composition. Table S3: Procheck statistics analysis of a predicted three-dimensional model of *Klebsiella pneumoniae* histidine decarboxylase. A. Ramachandran Plot statistics. B. G-Factors parameters. Table S4: Hydrophobic interactions between cinnamaldehyde and docked amino acid residues of *Klebsiella pneumoniae* histidine decarboxylase.
