# Supplementary material for: Anti-Allergic Potential of Cinnamaldehyde via the Inhibitory Effect of Histidine Decarboxylase (HDC) Producing Klebsiella pneumonia
Source: Molecules. 2020 Nov 27;25(23):5580. doi: 10.3390/molecules25235580 (PMC7730296; doi:10.3390/molecules25235580)
Supplement: Supplementary file 1 [file molecules-25-05580-s001.pdf]

Figure S1. In silico docking of Histamine, Histoidine and PLP for proof of evidence on the binding site of amino acid residues. A. Interactions between histamine and docked amino acid residues of *Klebsiella pneumoniae* histidine decarboxylase. B. Interactions between docked amino acid residues of *Klebsiella pneumoniae* histidine decarboxylase and histidine. D. Interactions between PLP and docked amino acid residue of in HDC of *Klebsiella pneumoniae*.

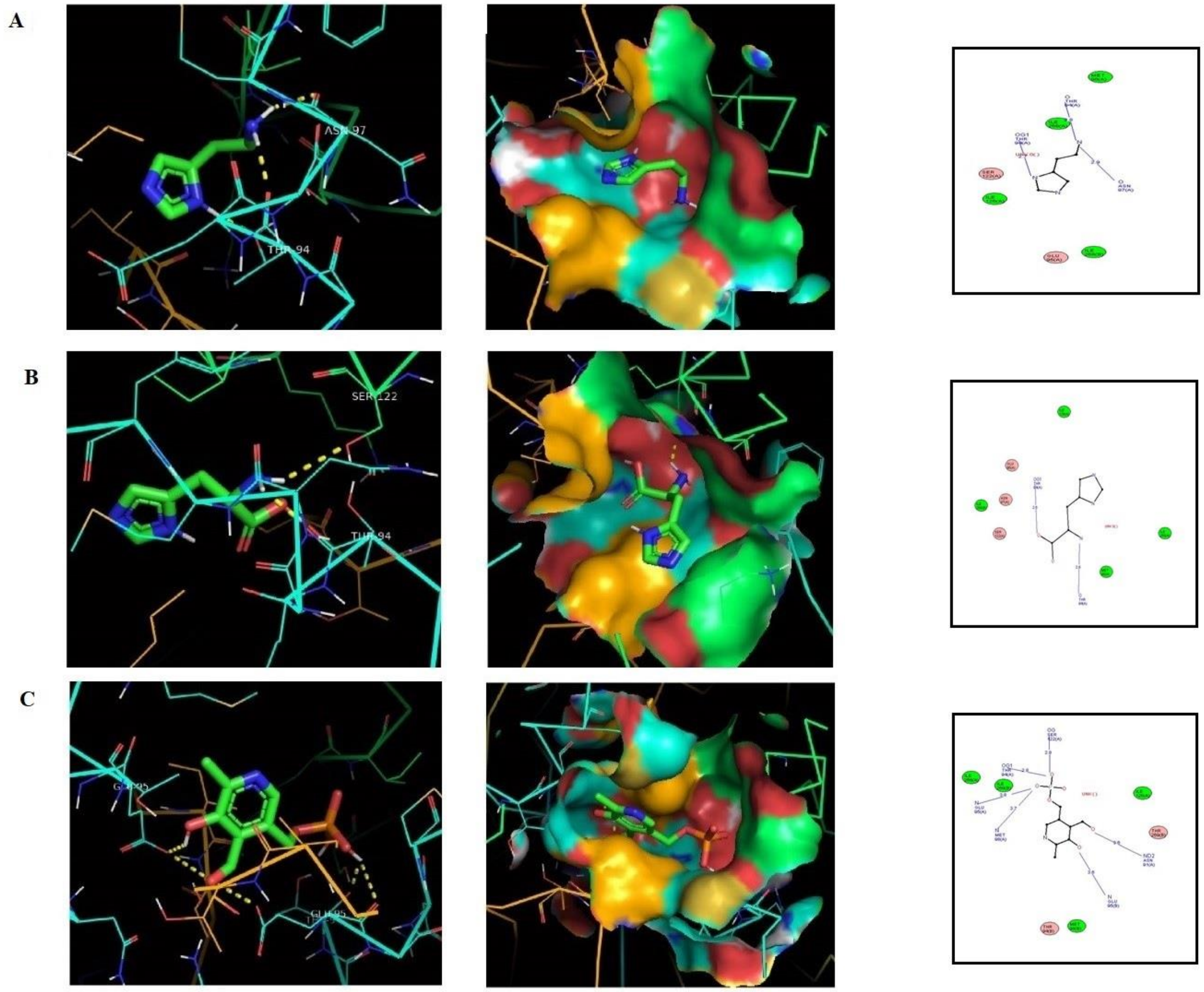

Figure S2. A. Differential screening medium for histamine production inoculated with Klebsiella pneumoniae; B. Dose dependent HDC inhibitory activity of CA on Differential screening medium for histamine production inoculated with Klebsiella pneumonia; C. Temperature stability study of CA and HDC inhibitory activity on Differential screening medium for histamine production inoculated with Klebsiella pneumonia; D. Inhibitory zone was determined using plate inhibitory method and plots was estimated by millimeter (mm).

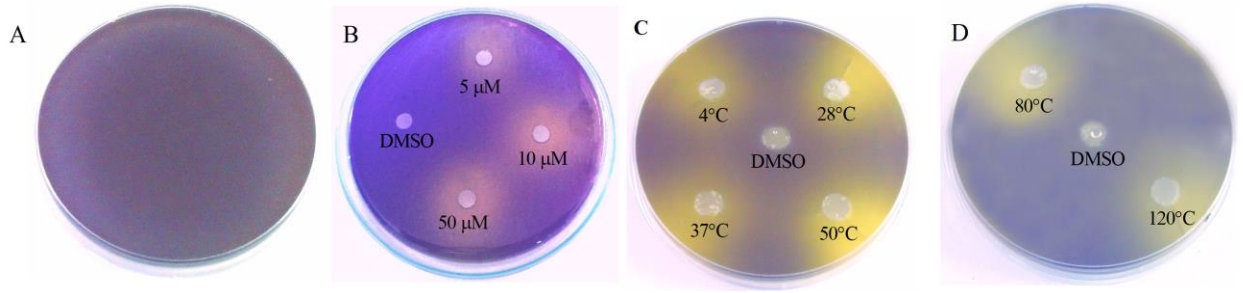

Figure S3. Microscopic examination of cell viability and survivability effect of CA on RBL-2H3 cells. Cell integrity and adhesion microscopic analysis by CA pre-incubation in response to DNP-BSA stimulation.

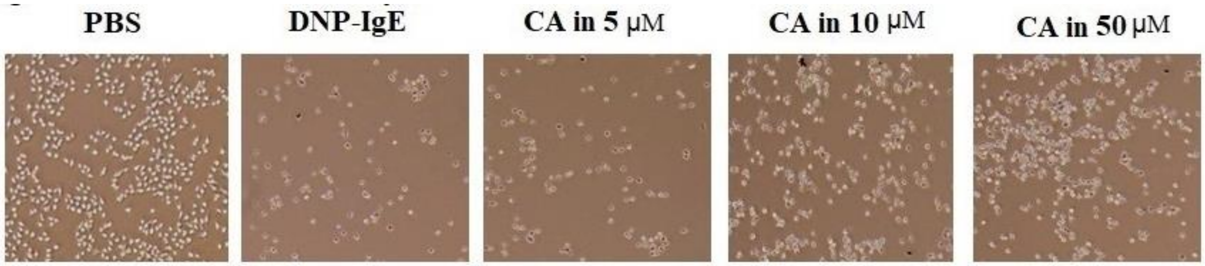

Table S1. *Klebsiella pneumoniae* histidine decarboxylase primary structural properties

Parameters		Theoretical Prediction
1.	Molecular weight (kDa)	43.52
2.	Isoelectric point	6.27
3.	Extinction coefficient (M^{-1} cm^{-1} at 280nm)	67310
4.	Estimated half-life (h):	
	Mammalian reticulocytes (in-vitro)	30
	Yeast (in-vivo)	>20
	Escherichia coli (in-vivo)	>10
5.	Instability index	33.43
6.	Aliphatic index	80.82
7.	Grand average of hydropathicity (GRAVY)	-0.304

Table S2. *Klebsiella pneumoniae* histidine decarboxylase Amino acid composition

Amino acid	composition	Percentage %
Ala (A)	21	5.5 %
Arg (R)	16	4.2 %
Asn (N)	20	5.3 %
Asp (D)	27	7.1 %
Cys (C)	8	2.1 %
Gln (Q)	11	2.9 %
Glu (E)	19	5.0 %
Gly (G)	23	6.1 %
His (H)	14	3.7 %
Ile (I)	37	9.7 %
Leu (L)	20	5.3 %
Lys (K)	25	6.6 %
Met (M)	9	2.4 %
Phe (F)	21	5.5 %
Pro (P)	16	4.2 %
Ser (S)	29	7.6 %
Thr (T)	16	4.2 %
Trp (W)	7	1.8 %
Tyr (Y)	19	5.0 %
Val (V)	22	5.8 %

Table S3. Procheck statistics analysis of predicted three dimensional model of *Klebsiella pneumoniae* histidine decarboxylase. A. Ramachandran Plot statistics. B. G-Factors parameters Ramachandran Plot statistics

Sl.No	Ramachandran Plot statistics	No. of residues (percentage)
1.	Most favoured regions [A,B,L]	273 (80.3 %)
2.	Additionally allowed regions [a,b,l,p]	51 (15.0 %)
3.	Generously allowed regions [~a, ~b, ~l, ~p]	10 (02.9%)
4.	Disallowed regions [XX]	6 (01.8 %)
5.	Total non-glycine and non-proline residues	340 (100 %)
6.	End-residues (excl. Gly and proline)	1
7.	Glycine residues	23
8.	Proline residues	16
9.	Total number of residues	380

(A) G-Factors parameters.

Sl.No	G-factor parameters	Score	Average score
1.	Dihedral angles:		
	Phi-psi distribution	-0.41	
	Chi1-chi2 distribution	-0.38	
	Chi1 only	0.13	
	Chi3 & chi4	0.67	
	Omega	-0.47	-0.28
2.	Main chain covalent forces:		
	Main chain bond length	-0.25	
	Main chain bond angles	-0.83	
			-0.59
	OVERALL AVERAGE		-0.38*

*Ideally, scores should be above -0.5. Values below -1.0 may need investigation

Table S4. Shows hydrophobic interactions between cinnamaldehyde and docked amino acid residues of *Klebsiella pneumoniae* histidine decarboxylase.

S/N	Ligand	Pubchem ID	Binding energy	Ligand efficiency	Intermole energy	Ligand atoms (ring)	Docked amino acid residue (bond length)
1.	Cinnamaldehyde	637511	-5.14	-0.51	-5.68	C-10'O<br>C-10'O	B chain PHE`99/HN (1.8 Å) (0.18 nm)<br>B chain GLY`96/O (2.6 Å) (0.26 nm)
2.	Histamine	774	-3.61	-0.45	-4.4	C-1′HN<br>C-4α′H1<br>C-4α′H2	A chain THR`94/OG1(1.7 Å) (0.17 nm)<br>A chain THR`94/O(1.9 Å) (0.19 nm)<br>A chain ASN`97/O (2.1 Å) (0.21 nm)
3.	Histidine	6274	-4.34	-0.39	-3.68	C-4α'COOH<br>C-4α′H2	A chain THR`94/O(2.1 Å) (0.21 nm)<br>A chain SER`122/OG (2.7 Å) (0.27 nm)
4.	Pyridoxal-5'-phosphate	1051	-3.71	-0.23	-5.76	C-5' OH<br>C-5' OH<br>C-4' H<br>C-4' H<br>C-3' OH	A chain THR`94/O (2.0 Å) (0.20 nm)<br>A chain GLU`95/O (2.8 Å) (0.28 nm)<br>A chain GLU`95/O (3.0 Å) (0.30 nm)<br>B chain GLU`95/OE2 (3.2 Å) (0.32 nm)<br>B chain GLU`95/OE2 (2.5 Å) (0.25 nm)
